# Clozapine-Induced Pneumonitis: A Case Report

**DOI:** 10.3389/fpsyt.2020.572102

**Published:** 2020-10-14

**Authors:** Tyler Torrico, Ronald O. Crandall, Carlos Meza, Sara Abdijadid

**Affiliations:** ^1^Department of Psychiatry, Kern Medical, Bakersfield, CA, United States; ^2^Department of Medicine, Ross University School of Medicine, Miramar, FL, United States

**Keywords:** adverse reactions, drug-induced, drug-induced lung disease, atypical antipsychotic, non- neutropenic fever, case report

## Abstract

**Introduction:** Clozapine is the most effective antipsychotic used for treatment resistant schizophrenia and recurrent suicidal behavior in schizophrenia or schizoaffective disorder. However, it has been underutilized due to its adverse reaction profile. Although clozapine is typically associated with neutropenia leading to increased risk of infection (i.e., pneumonia), there have been a few case reports of non-neutropenic, non-infectious drug-induced lung disease (i.e., pneumonitis). Although pneumonia and pneumonitis may have similar clinical presentation, their etiology, management, and treatment are different.

**Case presentation:** A 53-year-old African American female with schizoaffective disorder was hospitalized for being no longer able to appropriately utilize food, clothing, and shelter. The patient developed a sepsis-like presentation during clozapine titration which resolved after treatment for presumed pneumonia and clozapine discontinuation. When clozapine was resumed due to persistent psychosis, the patient again developed a sepsis-like presentation. Clozapine was again discontinued with no other interventions and the patient's symptoms resolved.

**Conclusions:** Drug-induced pneumonitis is a very rare adverse reaction of clozapine. Recognizing conditions that mimic sepsis may prevent patients from undergoing unnecessary laboratory testing and prevent exposure to unwarranted antibiotics.

## Introduction

Clozapine is a second-generation (also known as atypical) antipsychotic used for treatment resistant schizophrenia and for recurrent suicidal behavior in schizophrenia or schizoaffective disorder. Clozapine was the most effective at treating schizophrenic symptoms by a significant margin in a 2013 meta-analysis study that compared the effectiveness of 15 antipsychotic drugs (1). However, its use has been underutilized due to its adverse effect profile (2). Clozapine currently has boxed warnings for severe neutropenia, orthostatic hypotension/bradycardia/syncope, seizures, myocarditis/cardiomyopathy/mitral valve incompetence, and increased mortality in elderly patients with dementia-related psychosis. Its most common adverse reactions are generally cardiovascular (e.g., tachycardia, hypotension, hypertension), neuropsychiatric (e.g., drowsiness, sedation, dizziness, insomnia, vertigo) gastrointestinal (e.g., sialorrhea, constipation, nausea, vomiting), and/or weight gain (3).

There is less published information on the rarer adverse reactions of clozapine, especially lung disease. To our knowledge, seven case reports of non-infectious and non-allergic clozapine-induced lung disease have been reported in the literature (4–10). Clinical assessment for a drug-induced pneumonitis is challenging, as it is a diagnosis of exclusion and its clinical presentation may imitate sepsis. It is particularly challenging when clozapine is the offending agent which is also typically associated with neutropenia and increased risk of infection. In this case report, we document another case of clozapine-induced pneumonitis and discuss the clinical approach to this diagnosis as well as other important differentials that must be ruled out in acutely ill patients on antipsychotic medications. Consent to publish the case history was obtained from the patient. The Kern Medical Institutional Review Board approved the study protocol.

## Case Presentation

A 53-year-old African American female with a past psychiatric history of schizoaffective disorder, bipolar subtype, amphetamine use disorder, cannabis use disorder and multiple involuntary psychiatric hospitalizations, was admitted to the behavioral health unit on an involuntary legal hold after making grandiose and bizarre statements, and being no longer able to appropriately utilize food, clothing and shelter. Her home medications (daily dose) on admission were valproic acid 1,000 milligrams (mg), fluphenazine 20 mg, magnesium oxide 400 mg for migraine prophylaxis, diazepam 15 mg, diphenhydramine 50 mg, and trazodone 100 mg for insomnia. Past medical history was significant for conservatively managed hypertension, hypercholesterolemia, and diverticulosis. She has no known allergies to medications.

During week one of hospitalization, the patient's fluphenazine was replaced with olanzapine 30 mg due to persistent psychosis. Valproic acid was increased to 1,500 mg due to unstable mood and a sub-therapeutic serum level of 36 L (μg/ml). During week four of hospitalization the patient's psychosis remained refractory and clozapine titration was initiated while tapering off olanzapine. Complete blood count (CBC) was monitored closely during initial clozapine titration, with no signs of blood dyscrasias through day 9 of titration. On day 11 of clozapine titration (dose: 250 mg daily) the patient acutely developed sepsis like symptoms. The patient complained of chills and chest pain. The patient had a fever of 38.9°C; systolic and diastolic blood pressures ranging in the 90's and 60's respectively [mmHg]; tachycardia (140–160 beats per minute); tachypnea (up to 29 respirations per minute); and oxygen saturation on room air of 80%. On physical exam the appeared patient appeared diaphoretic, lungs were clear to auscultation, no abnormal heart sounds were heard, and no muscular rigidity, dystonia or other dyskinesias were present. Neurologic physical exam was grossly normal, no mental status change (patients orientation was unchanged from baseline psychosis), cranial nerves II-XII tested and intact, motor exam 5/5 throughout with no abnormal movements noted, sensory exam normal throughout. Initial laboratory studies were significant for white blood cell count (WBC) of 15.0 × 10^3^ (μL), absolute neutrophil count (ANC) of 13.3 × 10^3^ (μL), with no eosinophilia, and lactic acid level of 4.2 (mmol/L). Creatinine kinase (CK) was within normal limits at 65.0 μ/L. Other laboratory studies include a valproic acid level of 101.0 (μg/mL), unremarkable urinalysis, normal electrolytes, troponins and liver function tests. Eighteen-lead electrocardiogram was negative for signs of acute coronary syndrome.

**Figure 1:**
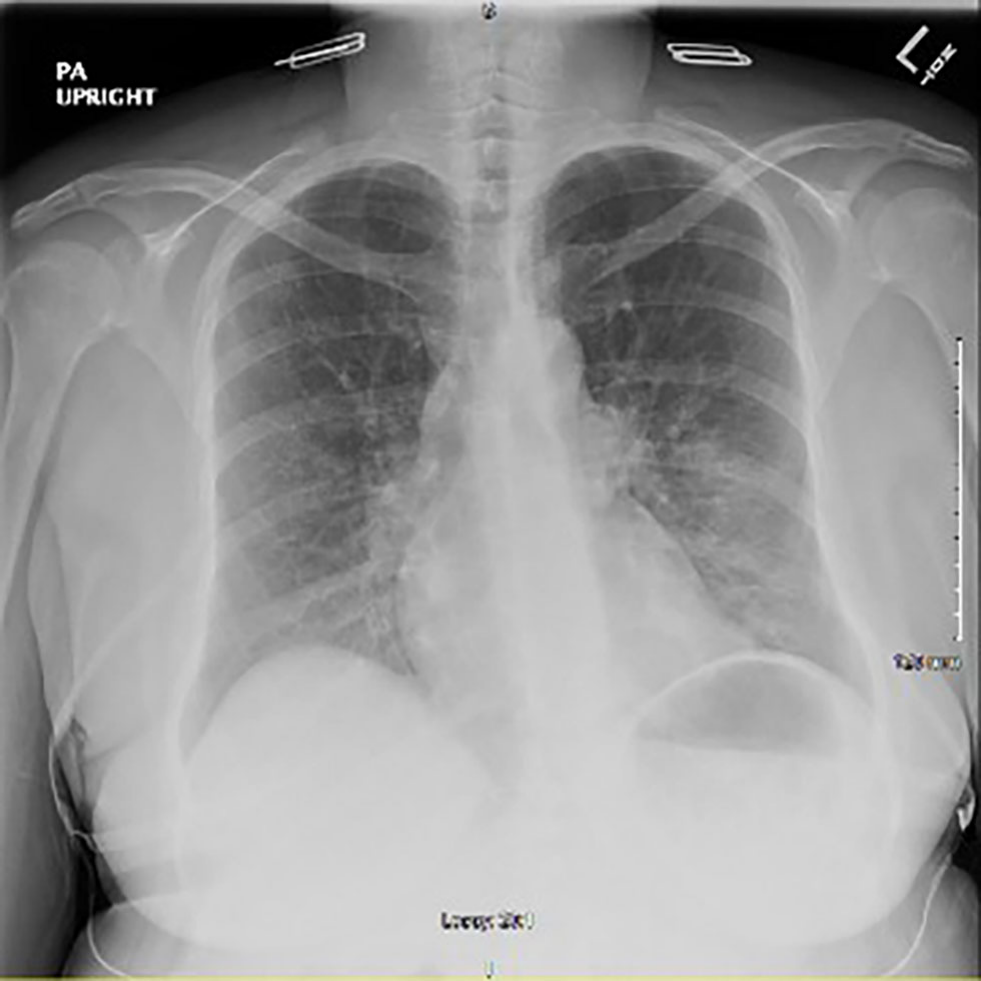
Initial chest x-ray after onset of symptoms showing peribronchial vascular opacities in the left lung base and left perihilar region.

Chest x-ray revealed peribronchial vascular opacities in the left lung base and perihilar region, raising concern for a developing pneumonia (Figure 1). Given the patient's septic presentation, intravenous (IV) vancomycin and piperacillin/tazobactam was started, along with acetaminophen for pyrexia. Although the patient had no evidence of neutropenia, clozapine was still discontinued out of concern for putting the patient at risk for neutropenia in the setting of a possible acute infection. After 24 h of holding clozapine and initiating treatment with broad spectrum antibiotics, the patient's vital signs stabilized, and leukocytosis resolved. The patient continued levofloxacin with presumed resolving pneumonia and restarted on clozapine 200 mg for her treatment resistant schizophrenia.

Within hours of restarting clozapine, the patient once again complained of chills and chest pain, but this time also had sialorrhea. Heart rate was 154, temperature of 39.3°C, respiration rate of 29 and oxygen saturation of 88%. The remainder of the physical exam was otherwise unchanged from the previous presentation, including no dyskinesias or muscle rigidity. The patient received nitroglycerin but had no symptomatic improvement and repeat electrocardiogram was still negative for signs of acute coronary syndrome. Blood and urine cultures continued to show no growth. Complete blood count was within normal limits, including WBC count of 7.2 × 10^3^ (μL) and ANC of 4.8 × 10^3^ (μL) with no eosinophilia. Repeat CK was not done as no neurologic and muscular abnormalities were noted and neuroleptic malignant syndrome was not suspected. Clozapine was held and the patient's symptoms resolved within 24 h without additional medical intervention.

**Figure 2:**
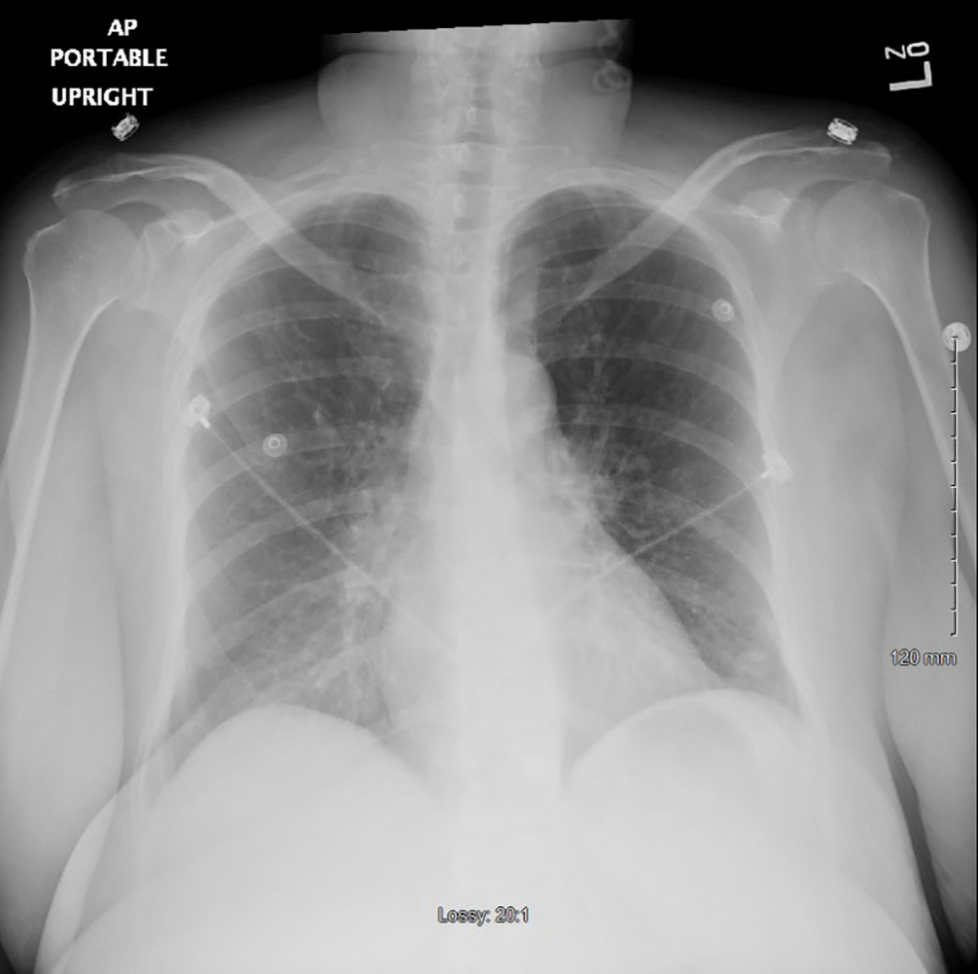
Chest x-ray 48 h after stopping clozapine (the second time) showing markedly improved lungs fields now almost completely clear.

A repeat chest x-ray 48 h after withholding clozapine showed markedly improved lungs almost completely clear of prior opacities (Figure 2). The patient was started on risperidone 2 mg and eventually paliperidone palmitate before being discharged, with no recurrence of fever or other signs of sepsis.

## Discussion

Drug-induced pneumonitis is a very rare adverse reaction of clozapine. To our knowledge, seven cases have been reported in the literature previously (4–10), but likely more cases have occurred unreported or undiagnosed. The clinical approach to clozapine-induced pneumonitis is a diagnostic challenge as neutropenia with increased risk of infection is far more commonly associated with clozapine than non-infectious lung injury (2). Still, clinicians should be mindful of the more rare but vast set of adverse reactions associated with the atypical antipsychotics, especially during the titration period.

The clinical presentation of clozapine-induced pneumonitis is nonspecific and has similarities to the clinical presentation of sepsis (particularly fever and tachycardia). It is essential to rule out infectious processes of lung injury as untreated these conditions may be life-threatening. Every prior case published that was reviewed of clozapine-induced pneumonitis was associated with pulmonary infiltrates on either chest x-ray and/or computed tomography (CT), while auscultation findings, symptom severity, oxygen saturation, and pulmonary function tests varied from case to case (4–10). Additionally, the diagnosis of clozapine-induced pneumonitis requires a temporal relationship between the onset of symptoms and the administration of clozapine, as well as resolution of symptoms and pulmonary infiltrates after clozapine is withdrawn. Applying the Naranjo adverse drug reaction probability scale may be helpful in assessing the likelihood of causality, particularly in cases such as this where the adverse reaction is demonstrated to be reversible and repeatable upon reintroduction to the suspected offending agent. This scale has four categories of drug reactions, doubtful, possible, probable, and definite, in the case report, a score of +6 was obtained, categorizing the likelihood of causality as probable (11).

Suspicion of drug-induced pneumonitis is generally not an indication for a lung biopsy or bronchoalveolar lavage when there is clinical improvement after holding the suspected agent (12). This is especially true when clozapine is the suspected agent as it has a relatively short half-life of about 14 h (2). However, in one similar case of clozapine-induced lung disease Arias et al. did perform a bronchoalveolar lavage which showed predominance of lymphocytes and a lung biopsy which revealed mild inflammation without granulomas (6). Hypersensitivity reactions have also been associated with clozapine (13, 14), however, specific laboratory makers such as eosinophilia have not been consistent in the presentation of clozapine-induced pneumonitis (4–10). There have been cases of clozapine associated eosinophilia causing multisystem organ failure, including pneumonitis, which may also present similarly to sepsis (10, 14).

In addition to ruling out infectious processes, cardiovascular pathology should be ruled out as well, as pulmonary embolism and cardiomyopathy both have been associated with clozapine use (15, 16). Finally, neuroleptic malignant syndrome (NMS) should always be ruled out in patients receiving antipsychotics who develop sudden changes to their physical health. NMS may occur at any time while a patient is receiving antipsychotic therapy, but it is most likely to occur within the first 2 weeks (17). Although the patient described in this case report was on day 11 of clozapine titration when she became acutely ill, her symptomology and lab work was not consistent with NMS. Specifically, there was no acute change in mental status, there was no muscular rigidity and therefore serum CK was also not elevated. Additionally, chest pain, shortness of breath, and pulmonary infiltrates are not suggestive of NMS. The patient did have some findings that are present in NMS, such as diaphoresis, autonomic instability (tachycardia), hyperthermia, and leukocytosis, but these are non-specific findings which are also present in sepsis. However, patients are more likely to develop NMS than a clozapine-induced pneumonitis, and therefore clinicians should always rule out extrapyramidal side effects and NMS when evaluating acutely ill patients on antipsychotic medications.

## Conclusion

Drug-induced pneumonitis is a very rare adverse reaction of clozapine. Clinicians should be mindful of the more rare but vast set of adverse reactions associated with the atypical antipsychotics, especially during the titration period when there is a temporal relationship to the onset of symptoms. Recognizing conditions that mimic sepsis may prevent patients from undergoing unnecessary laboratory testing and prevent exposure to unwarranted antibiotics.

## Data Availability Statement

The data displayed during the current study are not publicly available as they are part of protected health information of the patient described. But are available from the Kern Medical Center upon reasonable request and approval. Requests to access the datasets should be directed to tylertorrico@kernmedical.com.

## Ethics Statement

The studies involving human participants were reviewed and approved by Kern Medical Institutional Review Board. The patients/participants provided their written informed consent to participate in this study. We have obtained written, informed consent from the patient described in the case report for publication (including all data and images).

## Author Contributions

TT, RC, CM, and SA: wrote the manuscript. All authors contributed to the article and approved the submitted version.

## Conflict of Interest

The authors declare that the research was conducted in the absence of any commercial or financial relationships that could be construed as a potential conflict of interest.
